# A dataset of diversity and distribution of rodents and shrews in China

**DOI:** 10.1038/s41597-022-01422-2

**Published:** 2022-06-15

**Authors:** Jin-Jin Chen, Qiang Xu, Tao Wang, Fan-Fei Meng, Zhi-Wei Li, Li-Qun Fang, Wei Liu

**Affiliations:** 1 State Key Laboratory of Pathogen and Biosecurity, Beijing Institute of Microbiology and Epidemiology, Beijing, P. R. China; 2 Department of Epidemiology and Health Statistics, School of Public Health, Capital Medical University, Beijing, P.R. China

**Keywords:** Zoology, Infection, Agriculture

## Abstract

The rodents and shrews are important reservoirs for a large number of zoonotic pathogens. Here by performing a literature review, we determined the occurrence and distribution of rodents and shrews in China at three scales including province, city, and county levels. The peer-reviewed papers published in English and Chinese were collected, standard procedures were applied in reference books, field surveys and websites to remove duplicates, and information on recorded locations of rodents and shrews was extracted. The dataset contains 13,911 records of geo-referenced occurrences for 364 rodents and shrews distributed over 1,663 locations distinguished. As pathogens continue to emerge from rodents and shrews, this dataset could assist efforts to put preliminary bounds around a variety of spatial analyses of rodents and shrews, facilitate a better understanding of the transmission risk of the pathogens they carry, and be helpful for assessing the risk of future emergence of rodent-borne zoonoses.

## Abstract (summary)

**Table:** 

Measurement(s)	diversity and distribution of rodents and shrews
Technology Type(s)	literature review
Factor Type(s)	diversity and distribution
Sample Characteristic - Organism	rodents • shrews
Sample Characteristic - Environment	diversity • distribution
Sample Characteristic - Location	China

## Background & Summary

In the last decades, the incidence of human diseases caused by zoonotic viruses, bacteria, and parasites that are associated with small mammal reservoirs appears to have increased^1^. Rodents are the most abundant and diversified order of mammals, comprising approximately 42% of global mammalian biodiversity^2^, with an extensive range of species distributed in all continents except Antarctica^3^. Rodents and shrews include some small mammals, such as EULIPOTYPHLA, LAGOMORPHA, RODENTIA and SCANDENTIA^4–6^. They live in close contact with human beings, acting as a nexus among humans, domestic animals, pets, arthropod vectors (ticks, mites, fleas), and other wildlife, thus creating opportunity for rodents to transmit pathogens to humans, with hantaviruses, lassa viruses and *Yersinia pestis* as the prominent examples. Moreover, rodents can help to maintain pathogen transmission cycles in a number of different environments, varying from densely populated urban areas to rural areas and in the wilderness^7^. With an increased incidence and lack of effective prophylaxis or vaccines for most rodent-borne illnesses, a substantial burden remained on human health and agricultural economics in various countries^8^.

China is a country that hosts diverse climatic and ecosystems suitable for the propagation of a high diversity of rodents and shrews^9^. During the last decades we have seen a rise in human diseases that are associated with small-mammal reservoirs. There was relevant literature on rodent and rodent-borne pathogens in China^10^. However, the lately implemented policies that aim to protect, restore and conserve biodiversity in China, had resulted in ecologic changes promoting rapid increases in populations of rodents and shrews^11^. Other human activities and behaviors, including changing human settlement patterns (especially rapid urbanization in developed regions), the widespread migration of the human population, intensive trade activities, and explosive travel, are also enhancing the expanded transmission to human in new regions^12–14^. There is a great need to understand current knowledge on the diversity and distribution of rodents and shrews, so to attain a better understanding of their contribution to disease and a quantification of the risks per disease. This information is important for clinicians, researchers and professionals in the field.

The goal of this systematic review of published literature was to assembly a comprehensive database on the reports of contemporary occurrence and distribution of rodents and shrews in the mainland of China, and aggregate those indicators into a single open source dataset. We created a public database for rodents and shrews abundance and prevalence in China, reported from 1950 to 2021, encompassing 364 species of rodents and shrews in 1,660 counties and districts with 13,911 records. As rodent and shrew species-related records were better documented in Chinese, a study breaking language barriers for a more complete picture of spatial diversity in China and against a true baseline estimate of the geographic distribution of rodents and shrews would be useful for the new disease threats associated with rodent-borne zoonoses.

## Methods

### Data collection

**Fig. 1:**
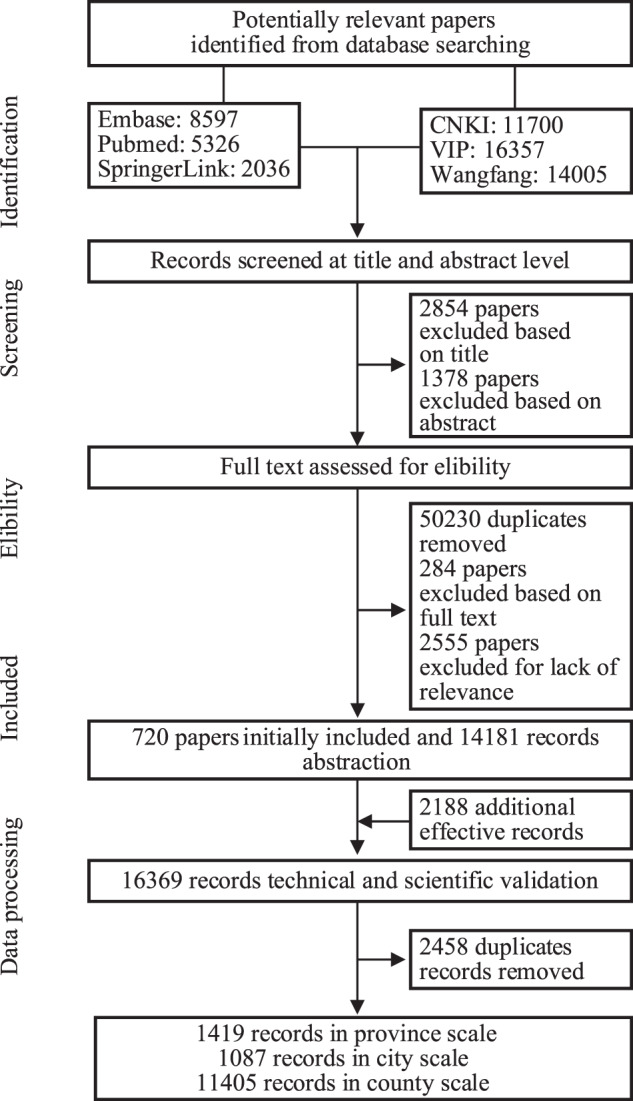
Flow chart of retrieval, screening, and inclusion of systematic database.

The protocols stated here have been adapted from previously published literature extraction efforts. A guide to our extraction has been included in Fig. 1, and shows the overarching process we followed to produce this dataset. We searched PubMed and ISI Web of Science for literature published in English, and China National Knowledge Infrastructure (CNKI), VIP database and Wanfang database as the core sources published in Chinese through the duration from 1950 to 2021. We used the following search terms: “Rodent”, “Mouse”, “Rat”, “Shrew”, and “China”, in combination with each of the 18 families of rodent and shrew species (either in English or in Chinese). A secondary manual search of the references cited in these articles was also performed to find relevant articles. For supplementation, the historical collection of rodent-related records that were documented in Chinese publications, with the multiple books (Fauna sinica, mammal, vol.6(2), cricetidae^15^, A guide to the mammals of China^16^, Distribution of mammal species in China^17^, Colored atlas of Chinese mammalians^18^, Mammalians of Tibet^19^, China’s red list of biodiversity: vertebrates, volume I, mammal^20^, Atlas of epidemiology of natural focus diseases in China^21^, Fauna of Beijing^22^, Fauna of Guizhou^23^, etc.), were also reviewed. Moreover, we standardize the species lists of the mammals of China from A guide to the mammals of China^16^ and Catalogue of mammals in China (2021)^24^.

Two researchers independently reviewed the information of each species of rodent and shrew, and entered the data into a standardized sheet to establish a database. Discrepancies were resolved by discussion between the two researchers and facilitated by a third senior researcher to reach consensus, and for those records where the time of discovery and location were missing in the articles, we contacted the corresponding authors for detailed information. The species lists were reviewed by specialists in small mammal groups^20^. Detailed descriptions for literature search and schematic procedures are provided in the Fig. 1.

**Fig. 2:**
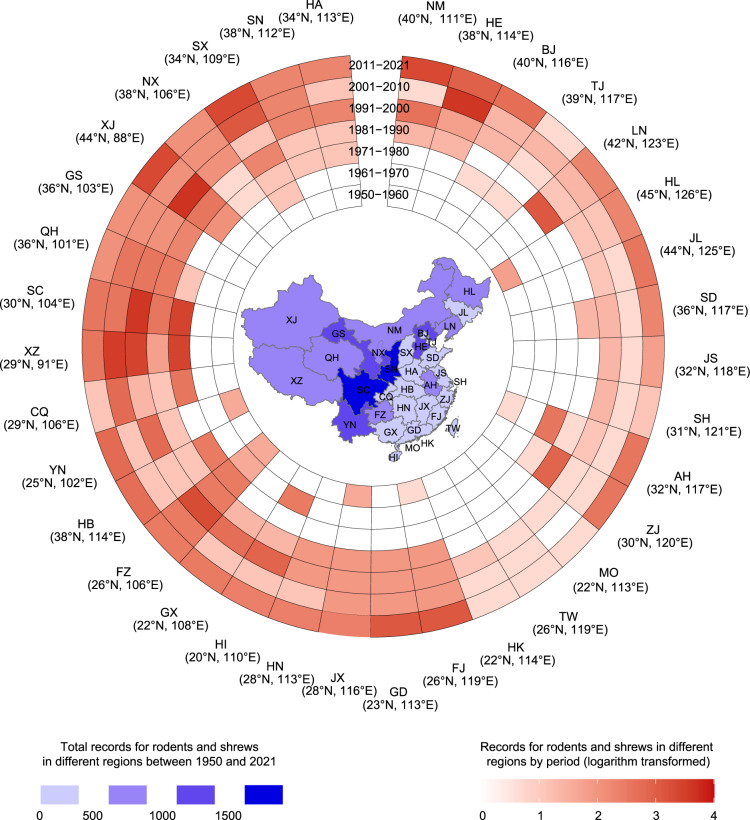
The number of reported records for rodents and shrews in different provinces between 1950 and 2021 is shown on the map of the mainland of China, Hong Kong, Macao, and Taiwan. The ring contains the data of records for rodents and shrews in other regions in 7 periods, and the latitude and longitude of the capital city of each province are shown. Note: the name of each province is following the national standard of China (GB/T 2260-2007) on the map.

Using the key words searching, a total of 58,021 papers were retrieved for screening, comprised of 15,959 papers from English database and 42,062 papers from Chinese database. For the second-step screening, the abstracts of all returned references were reviewed to exclude those reporting solely clinical case or laboratory data or reporting diagnostic development, without mentioning any specific rodent species. For the third step screening, we reviewed the full text of all the remaining 720 papers in detail, from which a total of 430 Chinese papers and 290 English papers were determined to be eligible for data extraction (Fig. 1). The earliest Chinese and English publications were published in 1958 and 1986, respectively, and an abrupt increase of literature with rodent and shrew records was seen after 1980 (Fig. 2). The key data were extracted from the obtained papers, books, field surveys and websites: (i) species name of rodents and shrews, (ii) the reported geographic location information at province, city and county levels, (iii) time of identification and reporting, (iv) source of papers, books, field surveys and websites. All the data were entered into an excel spreadsheet for downstream analysis. After the initial data entrance, a twice re-check by two persons was performed to correct errors and remove duplicates. Particularly, historical changes in taxonomy of rodents and shrews were considered and a standardized terminology was applied for the same species^24^. Where necessary, names of study sites that were historically used were updated to the current name. In total, 13,911 records were compiled, with several records within each province shown across the study years (Fig. 2).

### Geo-positioning

Location information was manually extracted at the highest resolution for each record of rodents and shrews, and then was categorized into three administrative levels such as province, city, and county. For a species, we only remain one record at the county level if various geographic locations (e.g., township, village) were reported within a county, and then we deleted the duplications for those records in the same county from different studies. For publications that reported neither specific administrative region nor coordinates, e.g., a scenic area or a mountain, we searched the coordinates for investigation sites by using Baidu Map (https://map.baidu.com/) and recorded their coordinates, which were linked to the county map by using the ArcGIS 10.7 software (ESRI Inc., Redlands, CA, USA) so as to gain the location information at county level. China administrative boundary maps of province, city and county (2015) were collected from the Resource and Environment Data Cloud Platform, Chinese Academy of Sciences (http://www.resdc.cn). A total of 13,911 records were identified including 1,419, 1,087, and11,405 records respectively at the province, city, and county level. A “Location level” field was used to accurately demonstrate the spatial resolution such as province, city or county for each record in our dataset. This classification allowed for separate extraction and usage at different spatial levels for various purposes. We used RStudio Version 1.4.1103 and ArcGIS 10.7 software to analyze and visualize the obtained geographic data statistically.

## Data Records

In this distribution and diversity dataset of rodents and shrews in China, as accessible from figshare^25^, each dataset row describes a distinct record (an occurrence of rodents and shrews in a specific location as described at a set time-point). The database contains the following fields:

1. **ID:** Unique identifier code of the database records.
2. **Species Authority:** The authority or author credited with the first formal use of the species scientific name.
3. **Order:** Identification of order of rodents and shrews.
4. **Family:** Identification of family of rodents and shrews.
5. **Genus:** Identification of genus of rodents and shrews.
6. **Species:** Identification of species of rodent and shrew.
7. **English Name:** English names of species of rodent and shrew.
8. **Chinese Name:** Chinese names of species of rodent and shrew.
9. **Synonyms:** Different names of species of rodent and shrew.
10. **China’s Red List of Biodiversity (2021):** The protection status assessment of species of rodents and shrews in the International Union for Conservation of Nature (IUCN) category.
11. **Assessment information:** The assessment information section includes the year assessed, assessors and reviewers of species of rodent and shrew.
12. **Assessment Criteria:** The assessment criteria is displayed for taxa assessed as threatened (i.e., taxa evaluated as Critically Endangered, Endangered or Vulnerable), and standards that are nearly met are displayed for Near Threatened taxa.
13. **Distribution Note:** The non-endemic and endemic of the species of rodent and shrew.
14. **Location level:** The geographic scale of location (1 = province level, 2 = city level, 3 = county level).
15. **Province:** Province level information of rodents and shrews reported location, including names of 34 provinces, autonomous regions and municipalities of China.
16. **City:** City level information of rodents and shrews reported location, including names of 304 prefectural-level cities or autonomous prefectures.
17. **County:** County level information of rodents and shrews reported location, including names of 1,660 counties.
18. **Province code:** Adopt 2015 China’s administrative division province name code.
19. **City code:** Adopt 2015 China’s administrative division city name code.
20. **County code:** Adopt 2015 China’s administrative division county name code.
21. **Longitude:** Longitude of reported sampling sites.
22. **Latitude:** Latitude of reported sampling sites.
23. **GPS (XX, YY):** The centroid longitude and latitude coordinates of reported sampling sites.
24. **Data source:** Details about whether location data was extracted from literature, field survey, website and book (1 = published paper in Chinese, 2 = published paper in English, 3 = published book in Chinese, 4 = published book in English, 5 = field survey data, 6 = website data).
25. **Reference publish time:** The year of reference publication identified for data extraction.
26. **Reference name:** The full name of references identified for data extraction.
27. **Native or exotic records:** The native or exotic records of the species of rodent and shrew.

## Technical Validation

Herein, this dataset contains 13,911 records extracted from 720 kinds of literature that were published between 1950 and 2021, as well as 2,188 additional effective records from Fauna sinica, mammal, vol.6(2), cricetidae^15^, A guide to the mammals of China^16^, Distribution of mammal species in China^17^, Colored atlas of Chinese mammalians^18^, Mammalians of Tibet^19^, China’s red list of biodiversity: vertebrates, volume I, mammal^20^, Atlas of epidemiology of natural focus diseases in China^21^, Fauna of Beijing^22^, Fauna of Guizhou^23^, Handbook of the mammals of the world. volume 8^26^, Mammal species of the world: a taxonomic and geographic reference. volume 3^27^, etc.

A senior investigator supervised the data entry performed by three data extraction analysts, two members for the records entrance and double check by each other, one member for the validation, emphasizing removing duplicate data, standardizing the formatting and naming conventions across the data. Due to the considerable period of the literature search over 70 years, the classification and naming of rodents and shrews have changed, and we have taken “Catalogue of mammals in China (2021)^24^ and A complete checklist of mammal species and subspecies in China: a taxonomic and the geographic reference”^28^ as the standard criterion for the naming conventions. Records were checked strictly to ensure accuracy and extraction criteria were met, following a previously described approach used in Herrera *et al*.^29^ and Meng *et al*.^30^. Data were checked strictly to ensure that accuracy and extraction criteria were met, similar to the method used in Zhang *et al*.^31^.

**Fig. 3:**
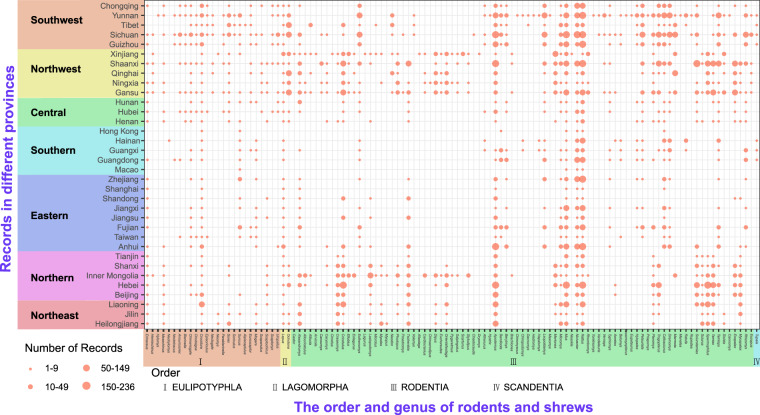
Number of genus records for different rodents and shrews at province level in the mainland of China.

**Fig. 4:**
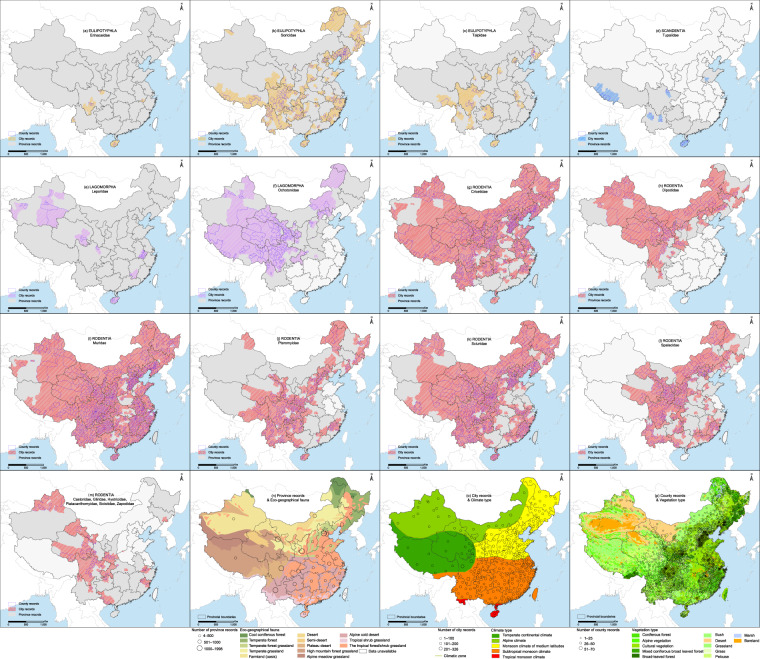
Locations of rodents and shrews occurrence of 18 rodent and shrew families most frequently reported rodents and shrews in the mainland of China, Hong Kong, Macao, and Taiwan province. The abundance of the database records varies with the multiple geographical and meteorological zones. (**a**) EULIPOTYPHLA Erinaceidae. (**b**) EULIPOTYPHLA Soricidae. (**c**) EULIPOTYPHLA Talpidae. (**d**) SCANDENTIA Tupaiidae. (**e**) LAGOMORPHA Leporidae. (**f**) LAGOMORPHA Ochotonidae. (**g**) RODENTIA Cricetidae. (**h**) RODENTIA Dipodidae. (**i**) RODENTIA Muridae. (**j**) RODENTIA Pteromyidae. (**k**) RODENTIA Sciuridae. (**l**) RODENTIA Spalacidae. (**m**) RODENTIA Castoridae, Gliridae, Hystricidae, Platacanthomyidae, Sicistidae, Zapodidae. (**n**) Province records with eco-geographical fauna. (**o**) City records with climate type. (**p**) County records with vegetation type.

The final georeferenced dataset contained 364 rodents and shrews in 4 orders, 18 families and 114 genera. We listed the catalogue, medical importance, and ecological characteristics of each kind of rodents and shrews in the Supplement Table 1, and data concerning medical importance and ecological characteristic were collected from “A catalogue and geographical distribution of the vectors of China^32^ and China’s red list of biodiversity: vertebrates, volume I, mammal”^20^. Figure 3 shows the diversity and number of records of all the rodents and shrews at the province level. Meanwhile, China has been divided into seven geographical regions, for each record, even though a rodents and shrews may be reported to be present in different locations, only one point was marked. As a result, the 18 rodent and shrew families were identified, and their distributions were illustrated in Fig. 4(a–m). The gray planes represent provincial records, the area covered by slash represents city records, and the light blue outline represent county records. The geographical locations have been color coded to represent the order of EULIPOTYPHLA, LAGOMORPHA, RODENTIA and SCANDENTIA. At the same time, the distribution density of rodents and shrews occurrences across different Eco-geographical fauna, climatic zones and vegetation zones are displayed and summarised in Fig. 4(n–p).

## Usage Notes

We provided a comprehensive and most updated comprehensive database for the geographic distribution and diversity of rodents and shrews at multiple scales in the mainland of China. The database contains 13,911 records, recording the distribution of 364 rodents and shrews that spanned from 1950 to 2021. Each record of the rodents and shrews was paired with relevant geo-positioning, thus enabling a future potential distribution prediction of rodents and shrews and disease risk assessment analysis.

Knowledge of the abundance and diversity of rodent reservoirs and hosts is crucial in supporting policy decision and directing the necessary actions to prevent and manage of relevant diseases. A large number of pathogens that are directly or indirectly transmitted by rodents are currently described. This dataset serves as the foremost comprehensive compilation of the distribution of rodents and shrews in the mainland of China. This comprehensive dataset can be applied in spatiotemporal dynamic investigations of rodents and shrews distribution at multiple geographical scales in China. Additionally, it can give a broad overview of the various diseases that humans may acquire from these rodents modelling their contribution to disease and quantifying of the risks per disease.

## Supplementary information

Supplement Table 1. Catalogue, medical importance and ecological character of 364 rodents and shrews in China (2021).

Rodent Database

## Data Availability

No custom code was made for the compilation and validation procedures in this dataset.
